# Gleanings from Our Exchanges

**Published:** 1873-10-15

**Authors:** 


Subcutaneous Injections.—Dr. Constantin Paul recommends glycerin as a dissolvent for subcutaneous injections. He considers it to be far superior to water, alcohol, etc.; it is neutral, can be kept easily, and is, of all liquids, the one which approaches the nearest to the composition of subcutaneous cellular tissue.—*Lancet*, Aug. 2, 1873.

Capillary Puncture of the Pericardium by Subcutaneous Aspiration.—In the *Lancet*, for March, 1873, we find a report, by Dr. Chairon, of a case of capillary puncture of the pericardium. The patient was a young soldier, who, at the end of an attack of pleurisy, presented all the symptoms of dropsy of the pericardium. M. Chairon introduced a capillary needle into the pericardial sac, and drew off a large quantity of sero-sanguinolent fluid, which quickly gelatinized. No accident attended the operation, and the following day he found the patient lounging about the passages of the hospital.

### Gleanings from Our Exchanges.

### ADVANTAGES OF CIRCUMCISION FROM A SURGICAL POINT OF VIEW.

Dr. Cadell read a paper on this subject before the Med. Chirurg. Soc., Edinburgh. He considered it in four aspects: 1. In infancy. 2. In boyhood. 3. In adult life. 4. In old age. He described:

1. The local and constitutional disturbance which may be set up by a long prepuce in infancy, and showed how these might be immediately relieved by circumcision. He read notes of a case, and also referred to those of Mr. Bryant, illustrating the effects of an adherent prepuce on the urinary organs, and the relief obtained by circumcision.

2. In boyhood, he believed that a long prepuce, by imprisoning the secretion from the glans, might be an exciting cause of masturbation; and if there was an hereditary disposition to nervous affections, epilepsy and insanity might be thereby induced.

3. In adult life, circumcision would facilitate cleanliness, diminish the secretion from the glans, so that the great cause of non-venereal excoriation would be removed, and thus render the mucous surface less susceptible to the venereal poison.

4. In old age, he cited Mr. Hey's opinion, that a congenital phimosis was an exciting cause of cancer in the penis.

In conclusion, Dr. Cadell remarked that he would strongly recommend circumcision in boys between infancy and puberty, whenever a congenital phimosis caused them the slightest inconvenience.

Prof. Lister said the cases alluded to by Dr. Cadell, of irritation caused by adherent prepuce, must be admitted to be of great interest. They knew that where adhesion existed there was often an accumulation of secretion, and they could understand that to be a cause of irritation. He should like to have it clearly brought out how far the symptoms in these cases were attributable to that cause, as distinguished from mere length of the prepuce. Though all would allow that cases of phimosis ought to be subjected to operation, it ought to considered whether circumcision was the best that could be done. The object could be obtained without mutilation. Mr. Jordan, of Birmingham, has written an interesting paper on the subject, showing that a perfectly natural condition of things might be obtained by the simple means of notching the ring of skin to the requisite extent, and then dividing the mucous membrane up to the *corona glandis*, and, avoiding all use of stitches, simply have the part drawn backwards and forwards twice every day. As regards the question of malignant disease, he might have been unfortunate, but he had now seen a large number of cases of cancer of the penis, not one of which was associated with the phimosis.

Dr. J. Bell said his experience in regard to circumcision was in cases of long standing and perfectly incurable nocturnal enuresis by small children who were in the habit of wetting the bed. In as many as four or five cases he had succeeded in effecting a perfect cure, by simply removing the redundant portion of the prepuce. In one case, a very bad case, a poor little fellow made his water first in the prepuce, which was like an orange at the end, and then he got rid of the water by squeezing it with his hand, the water coming out by a small aperture. That case was in George Watson's Hospital, and it became a question with the managers how to provide the necessary bedding for the boy. The operation performed was very simple, and was a complete cure. He (Dr. Bell) had very little experience of adherent prepuce; cases of adhesion of the prepuce were not so common as those of long prepuce.

Dr. Haliday Douglas said, that several years ago he was waited upon by a gentleman who had been married a few days before, and who had failed to effect connection. He was laboring under a very tight phimosis. He had never experienced any inconvenience during his life of twenty-five or twenty-eight years. He (Dr. Douglas) transferred him to Mr. Syme's hands, and, within twelve months, there were twins born to him. Another curious fact in this gentleman's history was this: In early life his brother had been relieved of phimosis, and three of his children, nephews of the first gentleman, had required to have the operation performed.

Dr. Watson was glad that the conclusion to which Dr. Cadell had arrived was, that where an elongated prepuce was a source of annoyance, it was right to relieve the person by removing it. As regarded the question of the comparative frequency of venereal complaints among persons who had been circumcised and those who had not, he might refer Dr. Cadell to a paper which appeared in the *Medical Times and Gazette*, 1st of December, 1855, by Mr. J. Hutchinson, in which it was shown that at the Metropolitan Free Hospital, situated in the Jews' quarter, in London, in the year 1854, the proportion of Jews to Christians among the out-patients was as *one* to *three*; at the same time, the proportion of cases of syphilis in the former to the latter was only as *one* to *fifteen*. Yet, that this was not the result of any higher degree of morality on the part of the Jewish population was obvious, because fully one-half of the cases of gonorrhœa occurred in Jews. This preventive influence of circumcision, as regards chancrous infection, led to hereditary syphilis being rarer among the children of Jews than of Christians. . . . . He was surprised that Dr. Cadell did not quote that greatest of all authorities on such matters, viz.: Dr. Ricord, who had said, in one of his published clinical lectures: "The prepuce is an appendix to the genital organs, the object of which I could never divine; instead of being of use, it leads to a great deal of inconvenience; and the Jews have acted kindly in circumcising their children, as it renders them free from one at least of the ills to which flesh is heir. The prepuce is, in fact, a superfluous piece of skin and mucous membrane which serves no other purpose than as a reservoir for the collection of filth, especially when individuals are inattentive to cleanliness." This was very strongly confirmatory of Dr. Cadell's views, though it appeared to Dr. Watson a little extreme.—*Edin. Med. Journal*, Feb. 1873.—*Western Lancet*.

The Easy, Safe and Radical Cure of Varicocele.—The *New York Medical Journal*, April, 1873, contains an article by Dr. J. F. Henstis, upon a new operation for varicocele, practiced by him. The important points are thus summed up:

1. A thorough emptying of the bowels immediately before the operation, so that the patient will not have to leave the recumbent posture for three days.

2. A passing of the wire behind the vein while the patient is standing, the vein being then swollen large, and easily defined and separated from the vas differens, etc.

3. A passing of the wire in front of the vein, with a blunt needle, while the patient is recumbent and the veins empty.

4. The administration of chloroform at the moment of tightening the ligature.

5. The preservation of the recumbent posture for three days, and the removal of the wire from around the vein, then administering chloroform to prevent pain.

6. The using of pure and very flexible silver wire, the leaden button and India rubber spring. The Doctor claims originality for keeping the patient in a recumbent posture, and so the veins empty for three days, a sufficient time to enable the plastic lymph to close the veins permanently.

The revenues of the London hospitals, including insane asylums, amount to £607,141 yearly.

Professional Risks.—We deeply regret to have to record the death of Dr. Pirrie, J. P., Consulting Physician of the Belfast General Hospital. This eminent physician must be counted amongst those who have fallen victims to the danger of professional life. His death was due to a wound by a speculum of bone, which happened to him in performing the operation of craniotomy. Two eminent professional men in London—Mr. Erichsen and Dr. Braxton Hicks—are at this time suffering from severe illness, passing on now, however, happily to cure, arising from wounds incurred in operating. Dr. Pirrie was a man of considerable accomplishment, and great local influence, and his premature death is largely felt and deeply lamented. He died at the age of 45.—*Brit. Med. Jour.*, Aug. 9, 1873.

The Cholera in Vienna.—The *Wiener Med. Presse*, July 6th, writes as follows: Our fears of the past several months have at last been sadly realized. The much dreaded cholera has again been observed during the past week, and has taken off more victims than before. Up to the 4th of this month there were seventeen cases, of which four died. But such a small number of cases can certainly not be reckoned to constitute an epidemic when our population is over a million souls, as is now the case. In order to prevent alarming and false reports, especially on the part of foreign journals, the Government has adopted the praiseworthy rule of publishing all the cases every day in the *Wiener Zeitung*. This official statement will quiet all apprehensions, it is to be hoped, while the Sanitary Committee secure all necessary regulations in the prevention of the spread of the disease.

Curability of Pulmonary Phthisis.—An article published by Dr. R. Massini, of Basle, in *Deutsch Arch. fur Klin. Med.* (H. 3 and 4, 1873), contains the following remarks on the above subject, the result of the author's personal experience: In pulmonary phthisis two-thirds of the fatal cases belong to cheesy pneumonia, and in only one-third of the cases is tubercle to be found. It is impossible in our present state of knowledge to assert that miliary tubercle is curable. It is shown by the results of post-mortem examinations (discovery of cicatrices in the apieces of the lungs) that cheesy pneumonia, when not complicated by tubercle, can be cured. Pulmonary phthisis, adds Dr. Massini, is frequently a consequence of abdominal typhus, and at Basle the mortality from phthisis has considerably diminished since the decrease of cases of abdominal typhus.—*London Lancet*, Aug. 23, 1873.

### BELLEVUE HOSPITAL, NEW YORK—NOTES OF TREATMENT.

*Intermittent Fever.*—Some prepartion containing quinine is usually given by all divisions in the treatment of this disease. The following, "Clark's Powder," is given almost exclusively on the third division:

℞.—Pulv. opii, gr. j;
Pulv. capsici, gr. iij;
Quinæ sulph., gr. x. M.
S.—Dose.

This is given about four hours previous to the time the chill is expected. If admitted during a paroxysm, or shortly after one has ceased, the powder is given and repeated as above. This rarely fails to break up even a prolonged series of paroxysms, and in recent cases almost invariably succeeds. By others, quinine alone is administered. The patient is rapidly brought under its influence in the following way: If the chill be expected in the morning, quin. sulph. gr. x are given the night previous, and again in the morning, one dose four hours, another dose two hours, before the time the chill is expected to occur. In the majority of cases this is succeessful in warding off the chill. If, however, it occur, a hypodermic of morphia is sometimes administered; but oftener the above dose (gr. x) is repeated in the hot stage, and quinism produced and maintained until the disease yields. In addition to this, some preparation of iron is given, as tri. ferri. chlorid. ♏x-xxx t. i. d. The hypodermic administration of quinine is being used extensively here, usually with favorable results. The solution adopted is the following, suggested by Dr. F. D. Lente, of Cold Spring, New York:

℞.—Quiniæ sulph. gr. l;
Acid sulph. dil., q. s.;
Aq. ebullient., ℥j.
Allow this to cool; then add—
Acid carbolic. (cryst.), gr. iv. M.

Of this, ♏x-xxx or more may be injected subcutaneously without danger of producing abscesses, such as commonly arise by the use of the etherial solution. Dr. Lente states that, although he has used it constantly in his practice, he has never seen an abscess caused by it; and a similar experience has attended its use here. This method is especially useful in cases of coma into whose causation malaria is suspected to enter; also in cases where it is necessary to bring the patient rapidly under the influence of the drug.

When quinine fails to arrest the paroxysms, arsenic is employed. A patient with malarial neuralgia of several weeks' duration had been treated with quinine in all the methods recommended, with no beneficial effect. Liq. potassæ arsenitis ♏viij t. i. d. put an end to the trouble in two days, the patient being discharged cured in ten days.

*Hyperpyrexia.*—If malarial, this is treated by quin. sulph. gr. v. q. 4 h.; and even in diseases in which no malarial element exists quinine is given. A temperature of about 104 deg. F. is usually treated by quinine as above, and by tinct. aconit. rad. (Fleming's) ♏j. q. ½ h. for three or four doses; then q. 1 h. In sthenic inflammation this is the most common method, and is usually successful. If the temperature rise above 104 deg. F., sponging the surface with water is employed, somewhat differently on different divisions; some preferring cold, others tepid, water. Several cases of insolation were treated on one division as follows: by means of an ordinary garden sprinkler, water, as hot as could be conveniently borne, was sprinkled over the body, an attendant on each side of the patient fanning the surface vigorously meanwhile. By this means the temperature in all cases rapidly fell, and did not show the same tendency to rise immediately that is observed when cold water is used. Ice-bags to the head, and cold-water injections into the rectum, were also used in some cases.

*Diphtheria of Wounds.*—Several cases have occurred during the past month in the lying-in-wards of diphtheria of wounds of the mucous membrane acquired during labor. The practice has been, in almost every case, to cauterize the surface with argent. nitrat. fus., and to apply cloths, moistened with "black-wash," to the wounds. In one case cauterization was adopted, and no other local application made, the parts being syringed out thrice daily with sol. acid carbolic (gr. x-℥j). Resolution quickly followed in the last, and in all the others except one, which was complicated with puerperal fever. In all cases of fœtid lochia, injections are employed either of the sol. acid carbolic, or infus. chamomil. —*Philadelphia Medical Times.*

